# Phase Equilibria and Interdiffusion in the Ternary System Epoxy Oligomer–Polysulfone–Alkyl Glycidyl Ether

**DOI:** 10.3390/polym16010130

**Published:** 2023-12-30

**Authors:** Artem D. Ponomarenko, Uliana V. Nikulova, Aleksey V. Shapagin

**Affiliations:** Frumkin Institute of Physical Chemistry and Electrochemistry Russian Academy of Sciences (IPCE RAS), 31, bld.4 Leninsky Prospect, Moscow 119071, Russia; ponomarenhik@gmail.com (A.D.P.); ulianan@rambler.ru (U.V.N.)

**Keywords:** DGEBA, epoxy resin, polysulfone, alkyl glycidyl ether, diffusion, ternary phase diagram

## Abstract

Phase equilibria, interdiffusion and structure in the initial uncured mixtures of epoxy oligomer–polysulfone–alkyl glycidyl ether were studied. Binodal curves were constructed on isothermal sections of the ternary phase diagram. Thermodynamic mixing parameters were calculated and spinodal curves were plotted. The interdiffusion coefficients of components, establishing the technological modes of mixing the components, were determined. To validate the phase diagram, the phase structure of mixtures, the composition of which at a temperature of 40 °C corresponds to heterogeneous and homogeneous regions, was studied.

## 1. Introduction

For many years, epoxy binders have occupied a leading position as matrices of polymer composite materials with high mechanical characteristics [1,2]. However, pure epoxides do not always meet the required values of physical and mechanical properties, especially crack resistance [3].

To increase crack resistance, epoxy oligomers are usually modified with elastomers [4] or rigid-chain thermoplastics [5,6]. At the same time, the introduction of elastomers reduces the glass transition temperature of the matrices, which negatively affects the heat resistance of the composite [7]. On the contrary, the modification of epoxy binders with rigid-chain thermoplastics leads to both an increase in crack resistance [8] and glass transition temperature (T_g_) [9]. Thus, thermoplastics, such as polysulfone (PSU) [10,11], polyethersulfone [12,13], polyetherimide [14,15], etc. [16,17,18], are widely used as modifiers for epoxy binders [19].

Modification of epoxy oligomers with polymers soluble in the initial state leads to the formation of a heterogeneous phase structure during the curing process [20], due to which an increase in physical and mechanical characteristics is achieved [21]. In this case, to obtain the required set of operational characteristics, it is fundamental to form the required type of phase structure. To predict the type of phase structure, it is necessary to have information about the phase equilibria in the initial uncured multicomponent polymer systems. In [20], phase equilibria in the PSU–epoxy oligomer system were studied, and it was shown that the components are completely compatible over a wide temperature and all concentration ranges. Another work [10] presents the types of heterogeneous structures of cured epoxy–polysulfone systems. In this case, the maximum values of crack resistance correspond to this type of phase structure as “interpenetrating phases”. Note that information regarding the physical and mechanical properties of epoxy–polysulfone mixtures with the “phase inversion” type of phase structure is very limited [8]. This is due to the fact that, despite the homogeneity of epoxy–polysulfone mixtures over the entire concentration range, the introduction of more than 20 parts by weight of PSU into the epoxy oligomer significantly increases the viscosity of the system, which complicates both the research and the use of such binders for impregnation of the fibrous filler of a composite material [22].

To reduce the viscosity of epoxy oligomers, active diluents are used, such as alkyl glycidyl ether (AGE) [23], furfuryl glycidyl ether (FGE) [24], and diethylene glycol with an epoxy group (DEG-1) [25]. Epoxy–polysulfone mixtures with the addition of an active diluent were studied in [26]. Due to the addition of FGE, the authors were able to prepare a mixture with a high content of PSU in an epoxy oligomer and measure crack resistance values. It was shown that the phase structure of the “phase inversion” type is characterized by maximum values of crack resistance. A study of the phase structure of cured compositions with different ratios of components showed that the addition of an active diluent has a significant effect on it. However, when explaining the structure of cured compositions, the authors limited the investigation of the phase equilibria of three bicomponent systems. The absence of isothermal sections of the ternary phase diagram did not allow for a comprehensive approach to predicting and regulating the phase structure in a multicomponent curing composition.

That is why the purpose of this work was to study phase equilibria and interdiffusion in the ternary system epoxy oligomer–polysulfone–alkyl glycidyl ether in a wide temperature range, as well as to construct isothermal sections of the ternary phase diagram, calculate interdiffusion coefficients and determine the parameters of critical points.

## 2. Materials and Methods

### 2.1. Materials and Systems

**Figure 1:**
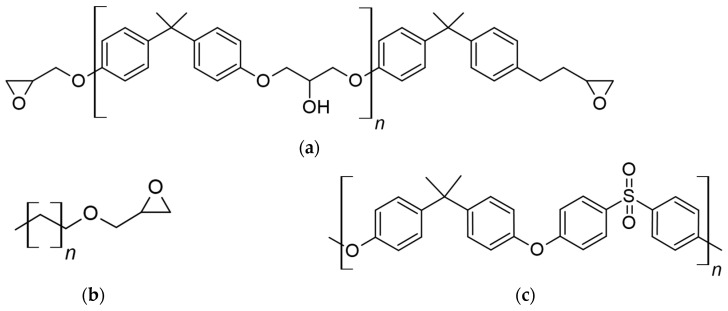
Structural chemical formulas for DGEBA (**a**), AGE (**b**) and PSU (**c**).

Epoxy oligomer DGEBA (CAS 25068-38-6, OOO SIPO, Moscow, Russia), active diluent alkyl glycidyl ether grade (AGE) c12-c14 (CAS 68609-97-2, OOO SIPO, Moscow, Russia) and polysulfone (PSU) PSK-1 (CAS 25135-51-7, AO NIIPM, Moscow, Russia) were used as research objects. The structural chemical formulas of the system components are shown in Figure 1, and the properties of the components are presented in Table 1. Table 2 presents the systems studied in the work.

Mixtures of DGEBA in AGE with different contents of components were obtained with a magnetic stirrer at a temperature of 50–60 °C for 1 h with a stirring speed of 800 rpm. PSU in DGEBA and in DGEBA/AGE mixtures was dissolved at 120–130 °C with periodic stirring for 3 to 8 h, depending on the PSU content. The ratio of components in the mixture, as well as the concentration of the second component/mixture on the phase diagram, are designated as φ and are presented in volume fractions.

### 2.2. Experimental Methods

#### 2.2.1. Optical Interferometry

Studies of solubility and mutual diffusion in binary mixtures, where the components are both individual substances and their mixtures, were carried out using the method of optical interferometry on an ODA-2 interferometer (IPCE RAS, Moscow, Russia) [27]. The light source was a laser with a wavelength (λ = 632 nm).

The method is based on obtaining the optical density distribution in the region where the components are combined under isobaric–isothermal conditions [28]. A 5 × 5 mm sample of PSU film or a DGEBA/PSU mixture was placed in a diffusion cell between glasses, on the inner surfaces of which a translucent layer of Ni–Cr alloy was deposited. The angle between the glasses was ≤2°, which ensured the formation of an interference pictures in the optics of the microscope. Then, the system was thermostated at a temperature above the glass transition temperature of the components, to ensure optical contact of the component, after which the space between the glasses was filled with DGEBA or a DGEBA/AGE mixture.

When studying diffusion zones in the DGEBA–AGE system, DGEBA was placed in the diffusion cell. After assembly, the cell was thermostated at 40 °C for at least 30 min and brought into contact with the AGE.

To obtain information about phase equilibria in systems, studies were carried out in the mode of stepwise heating and cooling with a step of 20 °C in the temperature range from 40 to 180 °C. At each stage, the system was thermostated to a state of equilibrium and the interference picture was recorded. The interdiffusion study was carried out in isothermal mode. The moment of contact of the components was considered the beginning of the process of interdiffusion.

**Figure 2:**
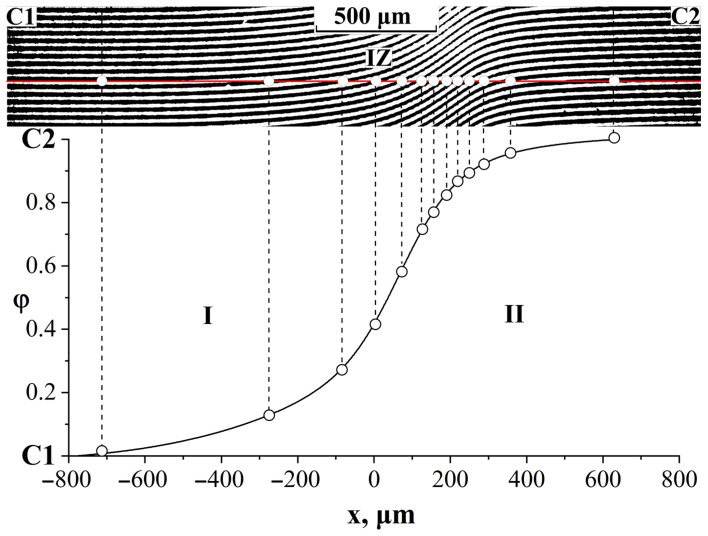
Methodology for constructing a concentration profile; IZ—interdiffusion zone; C1 and C2—areas of pure components; I—corresponds to the diffusion front of C2 into C1; II—diffusion front of C1 into C2.

The methods for processing interferograms of mutual diffusion zones, constructing phase diagrams, and determining interdiffusion coefficients did not differ from those described previously [16,29]. In the mutual diffusion zone, a line of equal thickness was drawn parallel to the interference fringes in the region of pure components and a concentration profile was constructed (Figure 2). The ratio C = 1/N, where N is the number of intersections of a solid line with interference fringes in the mutual diffusion zone, is equal to the increase in concentration when moving from one interference fringe to another. A continuous concentration profile characterizes fully compatible systems.

**Figure 3:**
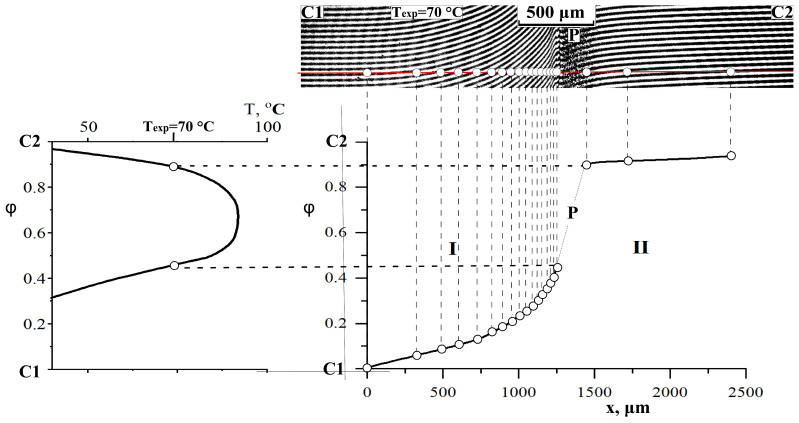
An example of constructing a phase diagram using optical interferometry; P—phase boundary; C1 and C2—areas of pure components; I—corresponds to the diffusion front of C2 into C1; II—diffusion front of C1 into C2.

In the interdiffusion zone, a phase boundary (P) is formed, which separates the areas of dissolution of the components from each other and creates a gap in the concentration profile (Figure 3). Knowing the number of lines in homogeneous solutions to the left and right of the phase boundary and calculating the total number of interference lines according to Equation 1 using the refractive indices of the components under study, we determined the concentration break points corresponding to the compositions of the coexisting phases [30,31].

The error in determining the compositions of coexisting phases at each temperature may vary slightly from system to system—this is due to the incremental increase in concentration for each band and depends on the total number of bands in the contact zone of the components, i.e., with differences in the refractive indices of the components. The error in determining concentrations lies in the range of 1.5–3%.

Experimental data on the compositions of the coexisting phases of the studied systems are presented in the accompanying data in Tables S2–S7.

#### 2.2.2. Refractometry

To calculate the compositions of coexisting phases of partially compatible systems in the temperature range under study using interference pictures, temperature dependences of the refractive indices of the components (initial substances and their mixtures) are required [30]. The difference in the refractive indices of the components of the diffusion system determines the total number of interference lines (N) according to the formula [27]:

(1) $$N=\frac{n_{1}-n_{2}}{△}$$

The increment of the refractive index per line (△) is determined by the characteristics of the cell (the angle between the glasses) and is 0.003.

The studies were carried out on an Abbe ATAGO NAR-2T refractometer (Atago Co., Ltd., Tokyo, Japan) in the “heating-cooling” mode in the temperature range from 20 to 120 °C with an accuracy of ±0.0001. The upper temperature limit is determined by the characteristics of the device.

A PSU film with α-bromonaphthalene liquid was placed between two prisms heated to 120 °C. Then, it was cooled to 20 °C in steps of 20 °C. The thermostatting time at each temperature was determined by the system reaching equilibrium and was at least 30 min.

The refractive indices of a mixture of DGEBA/PSU with different contents of substances, DGEBA, AGE and their mixtures were determined in the mode of stepwise heating and cooling from 20 to 120 °C without a special liquid providing optical contact.

Experimental data on the refractive indices of the studied systems are presented in the Supplementary Data in Table S1.

#### 2.2.3. Optical Microscopy

Qualitative in situ studies of the phase structure of the ternary system PSU–DGEBA–AGE with different contents of individual substances were carried out on an Olympus optical microscope BX51 (OLYMPUS, Japan). During the study, a sample of the mixture was cooled from 180 to 40 °C in steps of 5° and held at each step for at least 20 min. When signs of heterogeneity (turbidity of the solution) appeared, the temperature value and the optical image of the structure were recorded.

## 3. Results and Discussion

We have previously shown that, to predict the phase structure of curing systems modified with thermoplastics, information about phase equilibria in the original bicomponent systems is required [16,26,31]. In this work, the system is complicated by introducing an additional reaction component into it, which will require the study of phase equilibria in ternary systems. The solution to such a complex problem for polymer–oligomer–oligomer systems will involve the study of diffusion zones not only in binary systems of initial substances, but also in mixtures where one or both components are an oligomer–oligomer or an oligomer–polymer mixture. The analysis of diffusion zones will make it possible to determine the compositions of coexisting phases and construct isothermal sections of the ternary phase diagram.

### 3.1. Refractive Indices

**Figure 4:**
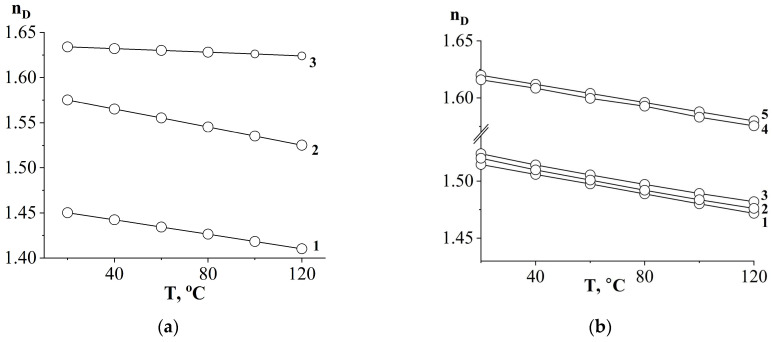
Temperature dependences of the refractive index: (**a**) initial substances: (1) AGE; (2) DGEBA; (3) PSU; (**b**) mixed compositions: (1) DGEBA50/AGE50; (2) DGEBA55/AGE45; (3) DGEBA60/AGE40; (4) DGEBA55/PSU45; (5) DGEBA50/PSU50.

To determine the compositions of coexisting phases using optical interferometry, information about the refractive indices of the system components is required. For this purpose, the temperature dependences of the refractive index (Figure 4) of PSU, DGEBA, AGE and their binary mixtures were obtained.

Since all measurements were carried out within the same physical state of the components of diffusion systems, the dependences have a linear form without breaks, which are characteristic of the transition through the glass transition temperature. Note that, with changes in the ratios of components in mixtures, the nature of the straight lines does not change. Only the absolute values of the refractive indices change. This is due to the homogeneity of the solutions presented in Figure 4b and the absence of phase and physical transitions in this temperature range.

**Figure 5:**
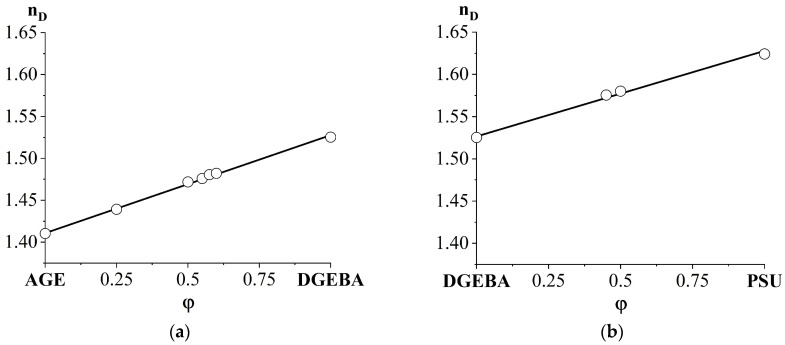
Concentration dependence of the refractive index of the AGE/DGEBA mixture (**a**) and DGEBA/PSU mixture (**b**) at a temperature of 120 °C.

The presence of one refractive index for mixed compositions indicates complete compatibility of the components. Also, the complete mutual solubility of its components is confirmed by the linear concentration dependence of the refractive index in the DGEBA/AGE and DGEBA/PSU systems at a temperature of 120 °C (Figure 5).

### 3.2. Phase Equilibria

The construction of a phase diagram involves the study of diffusion zones in binary gradient systems of initial substances, and, in the case of ternary systems, one or both components of the diffusion zone will be a homogeneous mixture of two initial substances. The maximum contact temperature for the components is 180 °C, which is due to the volatility of AGE at higher temperatures and changes in the concentration of the mixture component.

#### 3.2.1. Compatibility of Initial Substances

**Figure 6:**
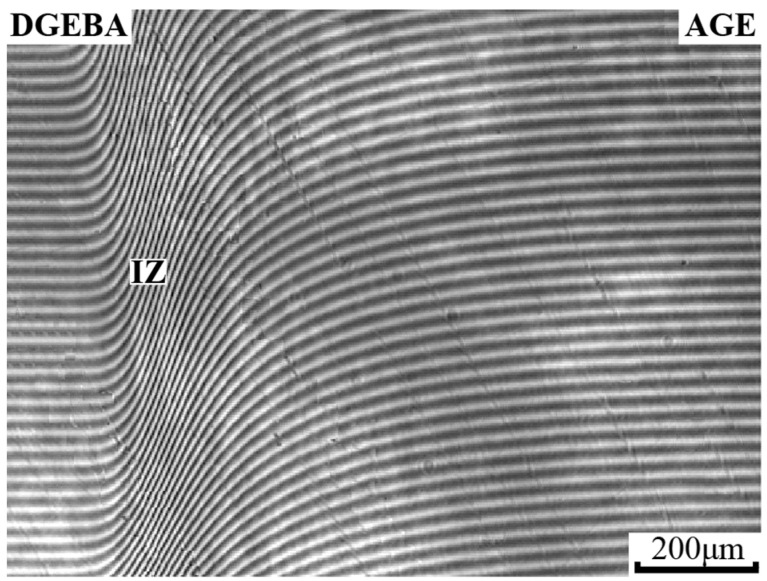
Interferogram of the interdiffusion zone of the DGEBA–AGE system at T = 180 °C, t = 16 min. IZ—interdiffusion zone.

**Figure 7:**
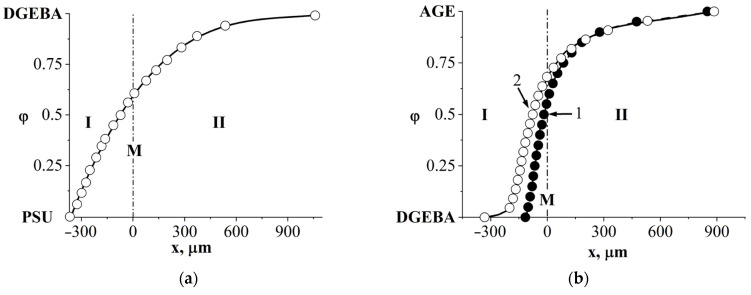
Concentration profiles of systems: (**a**) PSU–DGEBA at T = 180 °C, t = 16 min; (**b**) DGEBA–AGE at T = 180 °C, 1-t = 9 min, 2-t = 16 min. M—Matano–Boltzmann plane, I—corresponds to the movement of the diffusion front DGEBA in PSU and AGE in DGEBA, II—the diffusion front of PSU in DGEBA and DGEBA in AGE.

Figure 6 shows a typical interferogram of the diffusion zone of a fully compatible system using the example of a DGEBA–AGE mixture. It can be seen that all interference lines are identified and smoothly bend in the zone of interdiffusion of components. Based on the interferograms, the concentration profiles of the binary systems of the initial substances were constructed (Figure 7). It can be seen that throughout the entire gradient zone the concentration profiles are continuous, which characterizes the DGEBA–PSU (Figure 7a) and DGEBA–AGE (Figure 7b) systems as completely compatible throughout the entire concentration range at temperatures from 20 to 180 °C. Over time, the size of the diffusion zones increases (Figure 7b) in proportion to t^1/2^, which indicates the diffusion mechanism of mixing of components.

**Figure 8:**
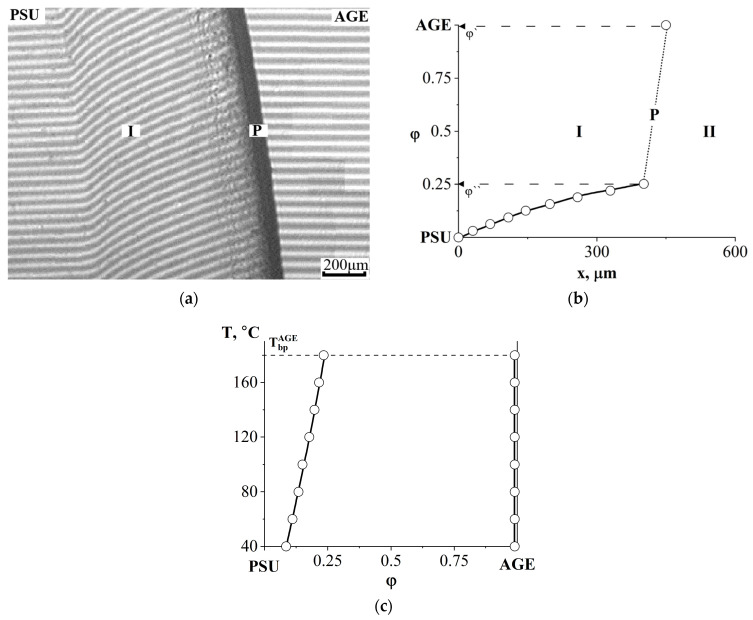
Interferogram of the interdiffusion zone of the PSU–AGE system at T = 180 °C and t = 16 min (**a**) and the corresponding concentration distribution profile (**b**), as well as the phase diagram of the PSU–AGE system (**c**). Т_bp_^AGE^—boiling temperature of AGE, P—phase boundary, I—corresponds to the movement of the AGE diffusion front in PSU, II—the PSU diffusion front in AGE.

The combination of semi-infinite PSU and AGE media is accompanied by the formation of a phase boundary (P) in the diffusion zone, separating the regions of dissolution of AGE in PSU and PSU in AGE (Figure 8a). A discontinuity appears in the concentration profile, which determines the compositions of the coexisting phases (Figure 8b). The concentration gap does not change with time, which indicates the equilibrium of a partially compatible system. With decreasing temperature, the nature of the distribution of concentrations in the zone of diffusion mixing of components is maintained, but the jump in concentrations at the interface increases. A phase diagram was constructed from the compositions of the coexisting phases (Figure 8c). It has been established that the PSU–AGE system is characterized by an amorphous separation phase diagram and belongs to the class of systems with upper critical solution temperature (UCST) [29,32,33].

#### 3.2.2. Compatibility of Binary Mixture with Polysulfone

**Figure 9:**
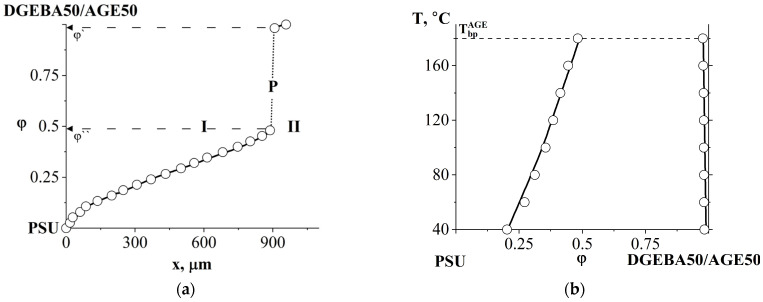
Concentration distribution profile of the PSU–DGEBA50/AGE50 system at T = 180 °C, t = 25 min (**a**) and phase diagram of this system (**b**). P—phase boundary, I—corresponds to the diffusion front of DGEBA50/AGE50 in PSU, II—diffusion front of PSU in DGEBA50/AGE50.

At the next stage, the construction of boundary curves of phase states inside the isothermal concentration triangles of the phase diagram is associated with the study of diffusion zones when contacting PSU with DGEBA/AGE mixtures (Table 2). The nature of the interferograms of PSU–DGEBA/AGE systems with an AGE concentration above 50% wt. did not differ from the PSU–AGE system (Figure 8). It has been established that the PSU–DGEBA/AGE system also belongs to the class of systems with amorphous stratification and is characterized by UCST (Figure 9).

**Figure 10:**
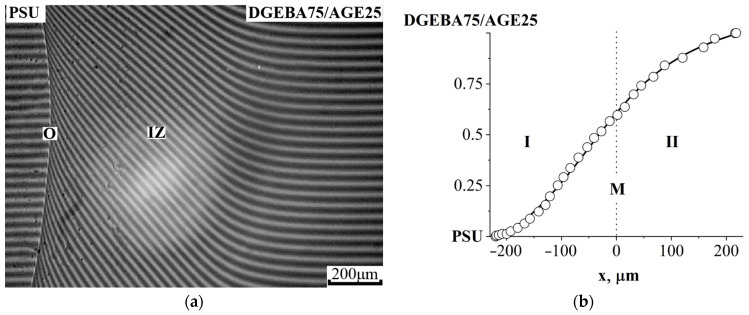
Interferogram of the interdiffusion zone of the PSU system—DGEBA75/AGE25 at T = 180 °C, t = 16 min (**a**) and concentration distribution profile at T = 180 °C (**b**). IZ—interdiffusion zone, O—optical boundary (explanation in the text), M—Matano–Boltzmann plane, I—corresponds to the movement of the DGEBA75/AGE25 diffusion front in PSU, II—the PSU diffusion front in DGEBA75/AGE25.

At an AGE concentration of 25% wt. the system is characterized by a continuous concentration profile, which indicates its complete compatibility (Figure 10). Note that, at the initial stage of dissolution, this system is characterized by the presence of an optical boundary (O) in the region of PSU-concentrated solutions, which is due to low interdiffusion coefficients in this concentration zone and, as a consequence, a high density of interference lines [32]. Over time, the diffusion zone expands and the intensity of blackening of the optical boundary gradually weakens until it completely disappears.

**Figure 11:**
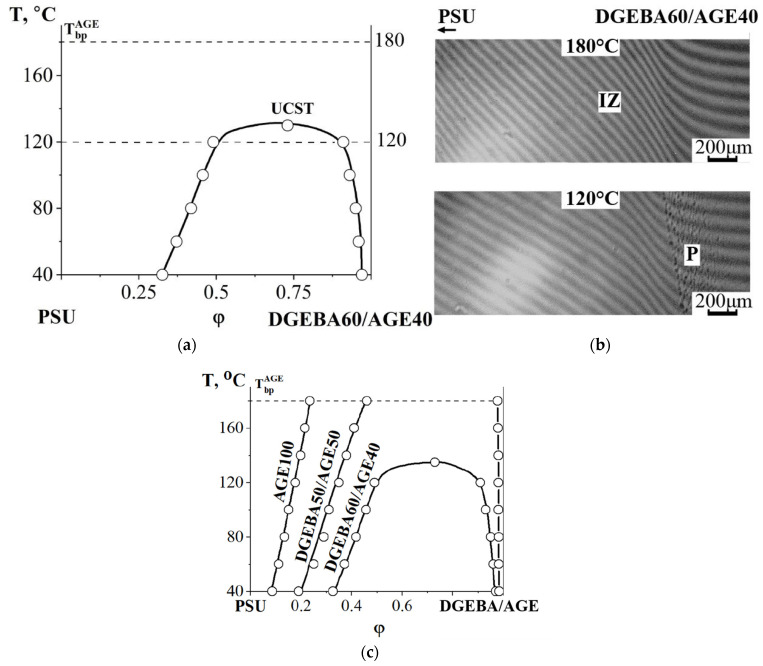
Phase diagram of the PSU system—DGEBA60/AGE40 (**a**) and interferograms of the interdiffusion zone at 180 °C and 120 °C (**b**); (**c**) binodal phase diagram curves of the PSU–DGEBA/AGE system at different AGE contents. T_bp_^AGE^—the boiling point of AGE, P—the phase boundary, IZ—the interdiffusion zone, M—the Matano–Boltzmann plane, I—corresponds to the movement of the DGEBA60/AGE40 diffusion front in PSU, II—the PSU diffusion front in DGEBA60/AGE40.

At an AGE concentration of 40 wt% in the PSU–DGEBA/AGE system (Figure 11a), we were able to fix the position of the critical point in the system (UCST 130 °C). This is confirmed by fragments of the interference pictures presented in Figure 11b. In Figure 11c, we generalized all the obtained binodal curves for the PSU–DGEBA/AGE system on the temperature–concentration field. It can be seen that, with increasing AGE concentration, the heterogeneous region of the phase diagram expands, and the UCST shifts to temperatures above 180 °C already at an AGE concentration of 50 wt%.

#### 3.2.3. Compatibility of Binary Mixtures

To clarify the position of the binodal of the ternary phase diagram of the PSU–DGEBA–AGE system, the diffusion zones of the PSU/DGEBA and DGEBA/AGE mixtures were studied (Table 2).

**Figure 12:**
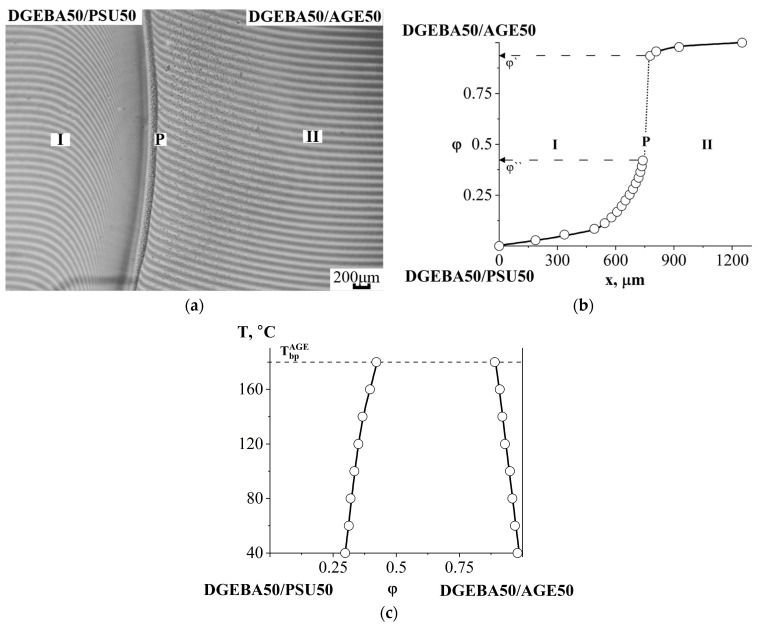
Interferogram of the interdiffusion zone of the DGEBA50/PSU50–DGEBA50/AGE50 system at T = 180 °C, t = 9 min (**a**), its concentration profile (**b**) and phase diagram (**c**). Т_bp_^AGE^—boiling point of AGE, P—phase boundary, I—corresponds to the movement of the diffusion front of DGEBA50/AGE50 in DGEBA50/PSU50, II—diffusion front of DGEBA50/PSU50 in DGEBA50/AGE50, φ^I^, φ^II^—compositions of coexisting phases.

Figure 12a shows an interferogram combining the semi-infinite media of the DGEBA50/PSU50 and DGEBA50/AGE50 mixtures. It can be seen that a phase boundary is formed in the diffusion zone, separating the areas of dissolution of the system components from each other. Based on the discontinuity in the concentration profile, the compositions of the coexisting phases were calculated (Figure 12b). As the temperature decreases, the concentration jump at the interphase increases, which classifies the system as a class of amorphous separation systems with UCST (Figure 12c).

**Figure 13:**
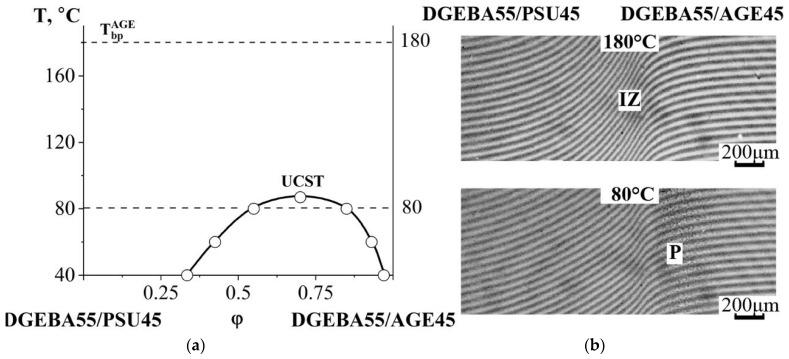
Phase diagram of the DGEBA55/PSU45–DGEBA55/AGE45 system (**a**) and interferograms of the interdiffusion zone at T = 180 °C and T = 80 °C (**b**). T_bp_^AGE^—the boiling point of AGE, P—the phase boundary, IZ—the interdiffusion zone, M—the Matano–Boltzmann plane, I—corresponds to the diffusion front of DGEBA55/PSU45 in DGEBA55/AGE45, II—diffusion front of DGEBA55/AGE45 in DGEBA55/PSU45.

Contacting the DGEBA55/PSU45 and DGEBA55/AGE45 mixtures (Figure 13) showed their complete compatibility in the temperature range from 85 to 180 °C. When the temperature dropped to 80 °C, a phase boundary formed. A further decrease in temperature was accompanied by an expansion of the heterogeneous region.

Qualitative results of studies of diffusion zones of binary systems, both initial substances and their mixtures, are presented in Table 3.

### 3.3. Ternary Phase Diagram

The information obtained above about phase equilibria in bicomponent systems, the components of which were both individual substances and their mixtures, was plotted on isothermal concentration triangles, the vertices of which correspond to individual substances, and the sides of the triangles are concentration scales.

**Figure 14:**
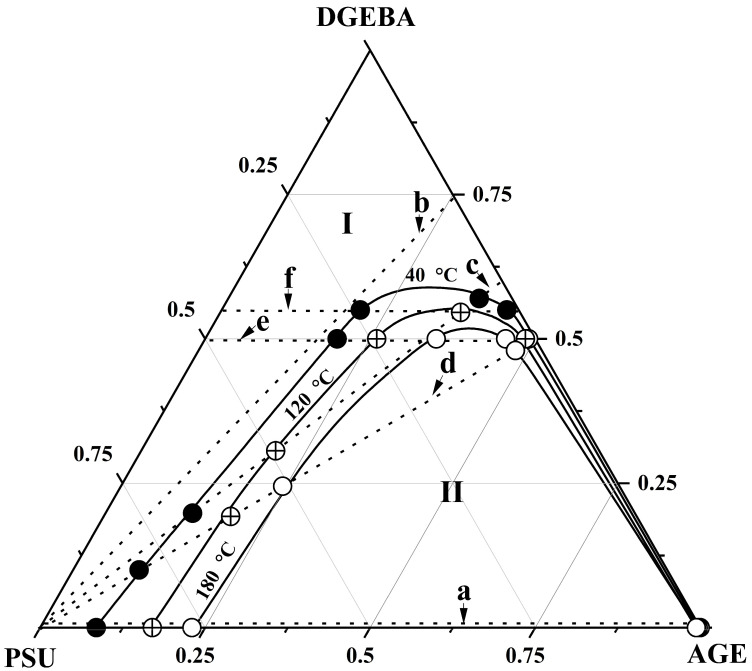
Isothermal sections of the ternary phase diagram of the DGEBA–PSU–AGE system at temperatures of 40, 120 and 180 °C. Solid lines are binodal curves. Dotted lines are the studied binary mixtures: (a) PSU–AGE, (b) PSU–DGEBA25/AGE75, (c) PSU–DGE-BA50/AGE50, (d) PSU–DGEBA60/AGE40, (e) DGEBA50/PSU50–DGEBA5/AGE50, (f) DGE-BA55/PSU45–DGEBA55/AGE45. I is the homogeneous region, II is the heterogeneous region. The different circles indicate the compositions of the coexisting phases of the studied systems in isothermal regimes.

Figure 14 shows a generalization of the binodal curves of the ternary system obtained at temperatures of 40, 120 and 180 °C on a triangular concentration field. The dotted line on the concentration field shows the bicomponent systems considered above, the compositions of the coexisting phases of which at given temperatures determined the binodal curves of the ternary system DGEBA–PSU–AGE. It can be seen that, with increasing temperature, the heterogeneous region of the phase diagram of the ternary system decreases on the concentration scale, which characterizes it as a system with UCST. Thus, the compositions of the coexisting phases of the ternary system DGEBA–PSU–AGE were obtained in the temperature range from 40 to 180 °C, which determine the binodal surface in the temperature–concentration field of the phase diagram.

**Figure 15:**
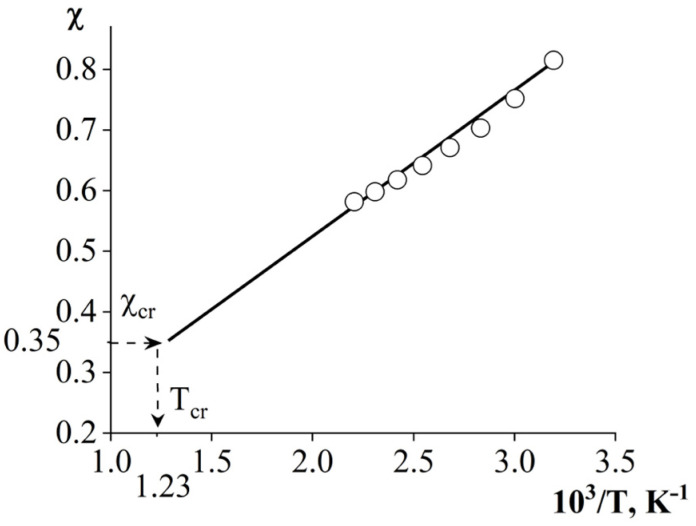
Temperature dependence of the Flory–Huggins interaction parameter for the PSU–AGE system. Definition of the UCST of a ternary system (T_cr_).

Since the temperature range of the study is limited by the thermal destruction temperatures of the components, it was not possible to experimentally determine the position of the UCST. In this regard, the position of the UCST of the ternary system in the temperature–concentration field of the phase diagram was calculated for the binary mixture with the lowest compatibility (PSU–AGE), using the Flory–Huggins theory of polymer solutions. The critical solution temperature (Figure 15) was calculated using the temperature dependence of the Flory–Huggins interaction parameter [29,33,34] (2) and its critical value (3).

(2) $$\chi_{cr}=\frac{\frac{ln(\frac{{\varphi_{1}}^{\prime \prime }}{{\varphi_{1}}^{\prime }})}{r_{1}}-\frac{ln(\frac{{\varphi_{2}}^{\prime \prime }}{{\varphi_{2}}^{\prime }})}{r_{2}}}{2({\varphi_{2}}^{\prime }-{\varphi_{2}}^{\prime \prime })}$$

(3) $$\chi_{cr}=\frac{1}{2}\left(\frac{1}{\sqrt{r_{1}}}+\frac{1}{\sqrt{r_{1}}}\right)$$

where ${\varphi_{1}}^{\prime }$, ${\varphi_{2}}^{\prime }$, ${\varphi_{1}}^{\prime \prime }$, ${\varphi_{2}}^{\prime \prime }$ are the compositions of the coexisting phases and $r_{1},\;r_{2}$ are the degrees of polymerization of the components. From Figure 15, it is clear that in the χ-(1/T) coordinates the dependence is linear. A decrease in the value of the pair interaction parameter with increasing temperature confirms that the PSU–AGE mixture is characterized by an amorphous separation phase diagram with UCST. It was established that the UCST of the ternary system is 536 °C. The critical concentration was determined using (4) [29,33], which follows from the Flory–Huggins theory of polymer solutions. The critical concentration of the ternary system is *φ*_cr AGE_ = 0.9 (Table 4).

(4) $$\varphi_{cr}=\frac{\sqrt{r_{1}}}{\sqrt{r_{1}}+\sqrt{r_{2}}}$$

**Figure 16:**
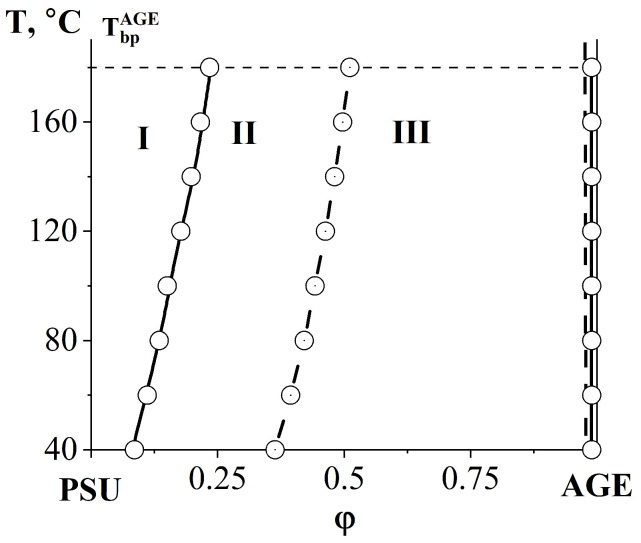
Phase diagram of the PSU–AGE system. Т_bp_^AGE^—boiling temperature of AGE, solid lines—binodal curve, dashed line—spinodal curve, I—homogeneous region, II—metastable region, III—labile structure region.

Spinodal curves (Figure 16) separating the regions of metastable and labile solutions are calculated using (5) of the Flory–Huggins theory [29,33]:

(5) $$\frac{1}{r_{1}\varphi_{1,S}}+\frac{1}{r_{2}\varphi_{2,S}}-2\chi =0$$

The thermodynamic characteristics of the ternary system were also determined using the Flory–Huggins theory of polymer solutions, modified by Tompa and Scott. To determine the critical point (plait point) [35], geometric analysis was used, which represented the joint fulfillment of two empirical conditions: Tarasenkov’s rule [36] and Alekseev’s diameter [37]. This approach was previously successfully tested in the work [38]. According to Tarasenkov’s rule, the extensions of all conodes beyond the triangle of rational compositions intersect at one point—the focal point (F). The Alekseev’s diameter is the midpoint of all conodes that lie on the same straight line intersecting the binodal at the critical point. In this case, the critical point on the phase diagram can be considered as a conode with zero length. Therefore, using this rule, we can state that the tangent to the binodal curve at the critical point also falls at the focal point.

**Figure 17:**
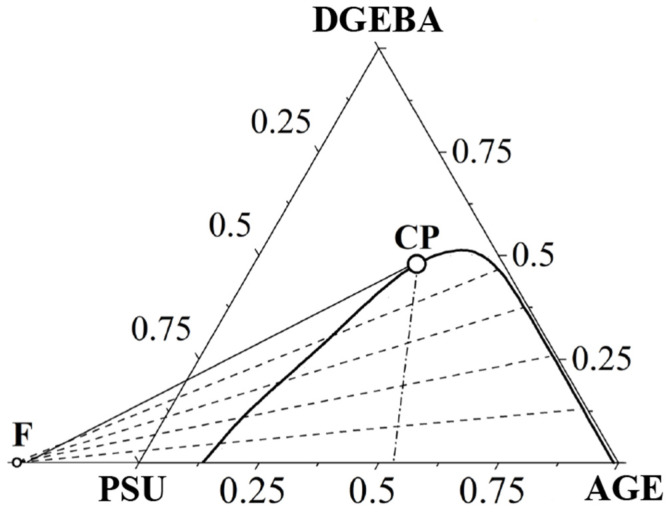
Determination of the critical point of the ternary system PSU–AGE–DGEBA at T = 180 °C. F—focus point, CP—critical point, solid line between F and CP—tangent to the binodal curve in CP. The dotted lines are conodes.

The joint fulfillment of the conditions described above is graphically presented in Figure 17 for an isothermal section of the phase diagram at a temperature of 180 °C. The values of critical concentrations determined by this method for the binodal curves of the ternary system at 40, 120 and 180 °C are presented in Table 4.

**Figure 18:**
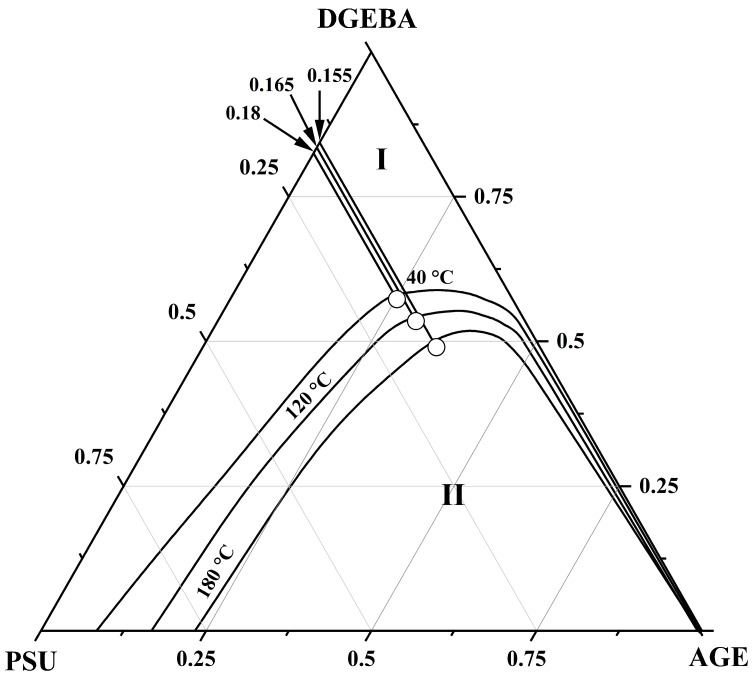
Phase diagram of the ternary system PSU–AGE–DGEBA at temperatures of 40, 120 and 180 °C. I—homogeneous region; II—heterogeneous region.

The position of the critical point and the focal point makes it possible to determine the compositions of coexisting phases for systems falling into the heterogeneous region of the ternary phase diagram (Figure 18).

### 3.4. Interdiffusion

**Figure 19:**
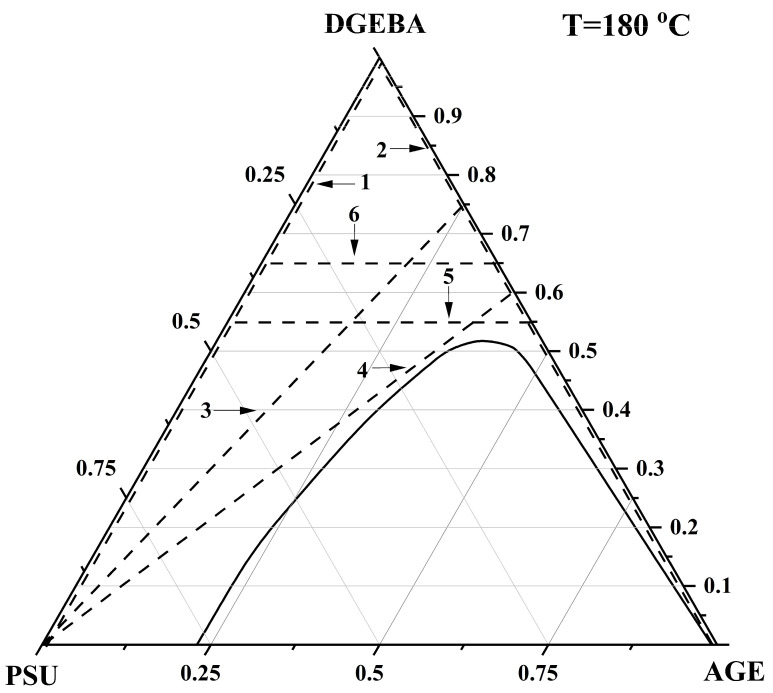
Phase diagram of the PSU–AGE–DGEBA system at T = 180 °C. The dotted line shows the studied systems: (1) PSU–DGEBA; (2) DGEBA–AGE; (3) PSU–DGEBA60/AGE40; (4) PSU–DGEBA75/AGE25; (5) DGEBA55/PSU45–DGEBA55/AGE45; (6) DGEBA65/PSU35–DGE-BA65/AGE35.

**Figure 20:**
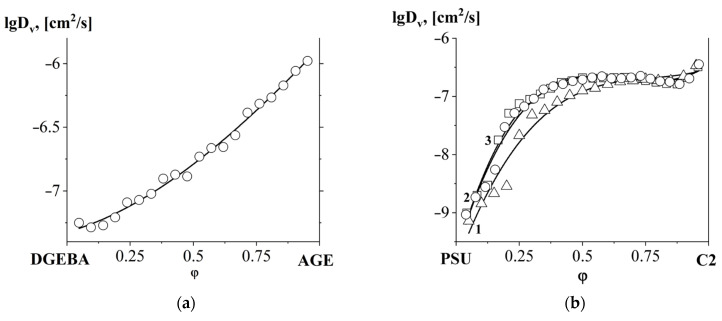

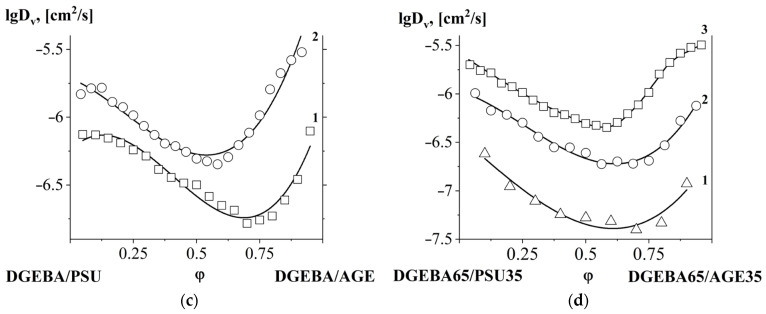
Concentration dependences of the interdiffusion coefficients of the systems: (**a**) DGEBA–AGE at 180 °C; (**b**) 1-△ PSU–DGEBA, 2-○ PSU–DGEBA60/AGE40, 3-□ PSU–DGEBA75/AGE25 at 180 °C; (**c**) 1-□ DGEBA55/PSU45–DGEBA55/AGE45, 2-○ DGEBA65/PSU35–DGEBA65/AGE35 at 180 °C; and (**d**) DGEBA65/PSU35–DGEBA65/AGE35 at temperatures: 1-△ 100 °C, 2-○ 140 °C and 3-□ 180 °C.

To develop technological regimes for the preparation of polymer composite materials based on the studied system, information on the macromolecular mobility of individual substances (interdiffusion) in the PSU–AGE–DGEBA system is important. Also, the values of the interdiffusion coefficients in the original ternary system are of fundamental importance for predicting the phase structure at the stage of curing of the modified reactive system. For mixtures located in the homogeneous region of the phase diagram at T = 180 °C (Figure 19), the concentration dependences of the interdiffusion coefficients were determined (Figure 20).

The mixing rate of the active diluent AGE with the main component of the epoxy binder DGEBA is characterized by the concentration dependence of the interdiffusion coefficient shown in Figure 20a. It can be seen that the nature of the dependence is additive, and the interdiffusion coefficients increase from DGEBA to AGE.

When PSU was contacted with DGEBA and DGEBA/AGE mixtures (Figure 20b), a sharp decrease in interdiffusion coefficients was observed from the region of PSU-enriched solutions. At the transition to the region of PSU-diluted solutions, an increase in interdiffusion coefficients was observed, which is associated with a decrease in the viscosity of the system due to an increase in the concentration of low molecular weight epoxides (DGEBA and AGE) in it.

Of greatest interest from fundamental and applied points of view are the concentration dependences of interdiffusion coefficients for mixed systems (Figure 20c). These curves are characterized by a minimum point in the region of average concentrations. As the content of PSU and AGE in mixtures increases, the minimum point shifts to the region of the DGEBA/AGE mixture. The decrease in interdiffusion coefficients and the shift in minimum concentration values are associated with the composition approaching the binodal curve in the concentration field of the ternary phase diagram.

**Figure 21:**
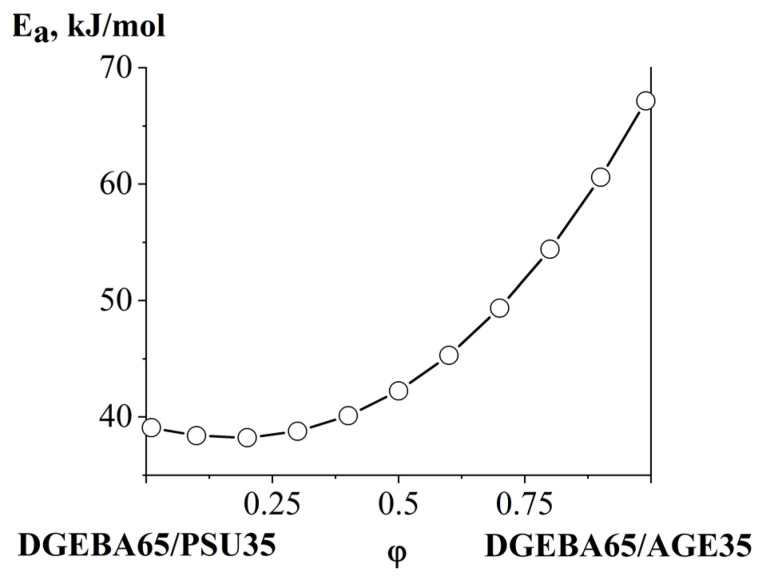
Concentration dependence of the apparent activation energy of interdiffusion of the DGEBA65/PSU35–DGEBA65/AGE35 system.

Interesting mixtures for applied applications in a homogeneous region with moderate concentrations of modifiers (PSU and AGE) were further studied in various isothermal modes (100, 140 and 180 °C). Based on the obtained concentration dependences of the interdiffusion coefficient (Figure 20d) using Equation (6) for the DGEBA65/PSU35–DGEBA65/AGE35 system, the apparent activation energy of interdiffusion was calculated (Figure 21).

(6) $$D_{v}=D_{0}{exp}^{\frac{{-E}_{a}}{RT}}$$

It was found that the apparent activation energy of interdiffusion with an increasing concentration of DGEBA65/AGE35 gradually increases from 40 to 70 kJ/mol. This nature of the dependence, despite the decrease in viscosity in the mixture with increasing AGE concentration and decreasing PSU content, is associated with a shift of the heterogeneous region of the PSU–AGE system in the region of solutions concentrated in AGE.

### 3.5. Optical Component Compatibility Studies

**Figure 22:**
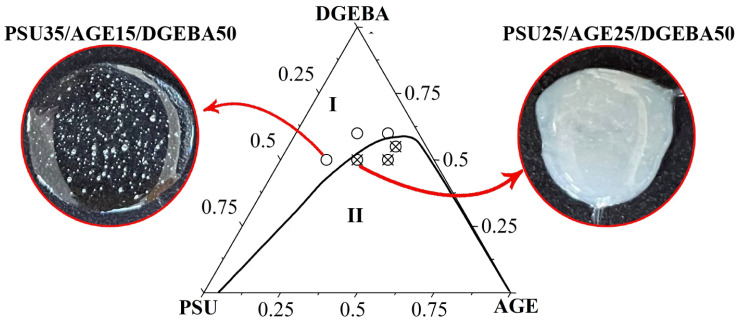
Phase diagram of PSU–AGE–DGEBA system at T = 40 °C: ○—compatible compositions; ⊗—incompatible compositions; compatibility of mixtures at T = 40 °C: (**a**) PSU 35/AGE15/DGEBA50; (**b**) PSU25/AGE25/DGEBA50; I—homogeneous area; II—heterogeneous region.

To confirm the position of the branches of the binodal curve, optical studies of the compatibility of the system components were carried out at T = 40 °C. The mixtures were prepared with a given composition (Figure 22) at T = 180 °C and cooled stepwise in steps of 5 °C, holding for at least 20 min. When signs of heterogeneity appeared, an image of the phase structure and the current temperature were recorded in an optical microscope.

The data obtained in situ confirm the correct position of the binodal surface on the temperature–concentration field of the phase diagram of the PSU–DGEBA–AGE system. The preparation of homogeneous PSU-concentrated mixtures was not possible due to the high viscosity of the system.

## 4. Conclusions

One of the ways to improve the performance characteristics of epoxy compounds is to modify them with thermoplastic polymers, which leads to an increase in the viscosity of the system and a deterioration in its technological characteristics. To reduce the viscosity of such systems, active diluents are used. The curing of such a complex system leads to the formation of heterogeneous structures that determine the physical and mechanical characteristics of the material.

In this work, phase equilibria in binary systems in which the components were individual substances (DGEBA, AGE and PSU) or their bicomponent mixtures were studied using optical interferometry. A ternary phase diagram of the amorphous separation of the PSU–AGE–DGEBA system was constructed in the temperature range 40–180 °C, characterized by a UCST equal to 536 °C. The critical points of binodal curves on the isothermal sections of the diagram are determined, which make it possible to calculate the compositions of coexisting phases. The concentration dependences of the interdiffusion coefficients in the homogeneous region of the ternary phase diagram have been studied. The influence of the modifying components (AGE and PSU) on the kinetics of the preparation of the three-component system was established. The apparent activation energy of the system in the concentration range most relevant for practical application was calculated. Its increase from 40 to 70 kJ/mol was shown with a decrease in the PSU concentration and an increase in the AGE content. In situ optical microscopy data confirmed the correct position of the binodal surface in the temperature-concentration field of the phase diagram of the PSU–DGEBA–AGE system.

Information about phase equilibria and interdiffusion in the PSU–AGE–DGEBA system is fundamental to predicting the phase structure during the curing process of a given system, which determines the set of operational characteristics. In the continuation of the work, we will study structure formation in this system, initiated by the chemical reaction of curing, and the influence of phase organization on the deformation and strength properties of cured systems.

## Figures and Tables

**Table 1:** Properties of research objects.

Object	Manufacturer	T_g_, °C	ρ, g/cm^3^	M_w_, Da
DGEBA	Sipo, Moscow, Russia	−18	1.15	380
AGE		−74	0.93	780
PSU	NIIPM, Moscow, Russia	183	1.24	35,000

**Table 2:** Systems studied in the work.

№	Component 1	Component 2
Mixtures of initial objects		
1	DGEBA	AGE
2	DGEBA	PSU
3	AGE	PSU
Mixtures of binary systems with PSU		
4	DGEBA75/AGE25	PSU
5	DGEBA50/AGE50	
6	DGEBA60/AGE40	
Mixtures of other binary systems		
7	DGEBA50/PSU50	DGEBA50/AGE50
8	DGEBA55/PSU45	DGEBA55/AGE45
9	DGEBA65/PSU35	DGEBA65/AGE35

**Table 3:** Compatibility results of the studied systems.

№	Component 1	Component 2	Compatibility
Mixtures of initial objects			
1	DGEBA	AGE	full
2	DGEBA	PSU	full
3	AGE	PSU	partial
Mixtures of binary systems with PSU			
4	DGEBA75/AGE25	PSU	full
5	DGEBA50/AGE50		partial
6	DGEBA60/AGE40		partial
Mixtures of other binary systems			
7	DGEBA50/PSU50	DGEBA50/AGE50	partial
8	DGEBA55/PSU45	DGEBA55/AGE45	partial

**Table 4:** Values of critical parameters of the studied systems.

Systems	Parameters	Values
PSU–AGE	χ _cr_	0.3636
	φ_cr AGE_	0.9
	T_cr_, °C	536
PSU–AGE–DGEBA at T = 180 °C	φ_cr PSU_	0.155
	φ_cr AGE_	0.35
	φ_cr DGEBA_	0.485
PSU–AGE–DGEBA at T = 120 °C	φ_cr PSU_	0.165
	φ_cr AGE_	0.3
	φ_cr DGEBA_	0.535
PSU–AGE–DGEBA at T = 40 °C	φ_cr PSU_	0.18
	φ_cr AGE_	0.26
	φ_cr DGEBA_	0.59

## Data Availability

The data presented in this study are available on request from the corresponding author.

## Supplementary Materials

The following supporting information can be downloaded at: https://www.mdpi.com/article/10.3390/polym16010130/s1, Table S1. Experimental data on the refractive indices of the studied systems. Tables S2–S7. Experimental data on the compositions of the coexisting phases of the studied systems.
