# Supplementary material for: Phase Equilibria and Interdiffusion in the Ternary System Epoxy Oligomer–Polysulfone–Alkyl Glycidyl Ether
Source: Polymers (Basel). 2023 Dec 30;16(1):130. doi: 10.3390/polym16010130 (PMC10780338; doi:10.3390/polym16010130)
Supplement: Supplementary file 1 [file polymers-16-00130-s001.zip › polymers-2756457-supplementary.pdf]

Table S1. Experimental data on the refractive indices of the studied systems.

Data set

Refractive index								
T, °C	PSU	DGEBA	AGE	DGEBA50/AGE50	DGEBA60/AGE40	DGEBA55/AGE45	DGEBA50/PSU50	DGEBA55/PSU45
20	1.6341 ± 0.0005	1.5752 ± 0.0005	1.4504 ± 0.0005	1.5145 ± 0.0005	1.5241 ± 0.0005	1.52 ± 0.0005	1.62 ± 0.0005	1.616 ± 0.0005
40	1.6321 ± 0.0005	1.5652 ± 0.0005	1.4424 ± 0.0005	1.5059 ± 0.0005	1.5142 ± 0.0005	1.5098 ± 0.0005	1.612 ± 0.0005	1.6086 ± 0.0005
60	1.6301 ± 0.0005	1.5552 ± 0.0005	1.4344 ± 0.0005	1.4976 ± 0.0005	1.5055 ± 0.0005	1.5009 ± 0.0005	1.604 ± 0.0005	1.5997 ± 0.0005
80	1.6281 ± 0.0005	1.5452 ± 0.0005	1.4264 ± 0.0005	1.4888 ± 0.0005	1.4971 ± 0.0005	1.4921 ± 0.0005	1.596 ± 0.0005	1.5929 ± 0.0005
100	1.6351 ± 0.0005	1.5352 ± 0.0005	1.4184 ± 0.0005	1.4802 ± 0.0005	1.4892 ± 0.0005	1.4837 ± 0.0005	1.588 ± 0.0005	1.5833 ± 0.0005
120	1.6241 ± 0.0005	1.5252 ± 0.0005	1.4104 ± 0.0005	1.4719 ± 0.0005	1.4821 ± 0.0005	1.4761 ± 0.0005	1.58 ± 0.0005	1.5756 ± 0.0005

Table S2-7. Experimental data on the compositions of the coexisting phases of the studied systems.

Phase diagrams of bicomponent systems

PSU-AGE				
T, °C	Left		Right	
	φ_{PSU}	φ_{AGE}	φ_{PSU}	φ_{AGE}
40	0.09 ± 0.015	0.91 ± 0.015	0.99 ± 0.015	0.01 ± 0.005
60	0.11 ± 0.015	0.89 ± 0.015	0.99 ± 0.015	0.01 ± 0.005
80	0.13 ± 0.015	0.87 ± 0.015	0.99 ± 0.015	0.01 ± 0.005
100	0.16 ± 0.014	0.84 ± 0.014	0.99 ± 0.014	0.01 ± 0.005
120	0.18 ± 0.014	0.82 ± 0.014	0.99 ± 0.014	0.01 ± 0.005
140	0.20 ± 0.014	0.80 ± 0.014	0.99 ± 0.014	0.01 ± 0.005
160	0.22 ± 0.013	0.78 ± 0.013	0.98 ± 0.013	0.02 ± 0.01
180	0.23 ± 0.013	0.77 ± 0.013	0.98 ± 0.013	0.02 ± 0.01

PSU-DGEBA50/AGE50				
T, °C	Left		Right	
	φ_{PSU}	$\varphi_{DGEBA50/AGE50}$	φ_{PSU}	$\varphi_{DGEBA50/AGE50}$
40	0.2 ± 0.023	0.8 ± 0.023	0.98 ± 0.023	0.02 ± 0.01
60	0.29 ± 0.023	0.71 ± 0.023	0.98 ± 0.023	0.02 ± 0.01
80	0.33 ± 0.022	0.67 ± 0.022	0.98 ± 0.022	0.02 ± 0.01
100	0.36 ± 0.02	0.64 ± 0.02	0.98 ± 0.02	0.02 ± 0.01
120	0.38 ± 0.02	0.615 ± 0.02	0.98 ± 0.02	0.02 ± 0.01
140	0.40 ± 0.019	0.59 ± 0.019	0.98 ± 0.019	0.02 ± 0.01
160	0.44 ± 0.019	0.56 ± 0.019	0.98 ± 0.019	0.02 ± 0.01

180	0.49 ± 0.018	0.51 ± 0.018	0.98 ± 0.018	0.02 ± 0.01

PSU-DGEBA60/AGE40				
T, °C	Left		Right	
	φ_{PSU}	$\varphi_{DGEBA60/AGE40}$	φ_{PSU}	$\varphi_{DGEBA60/AGE40}$
40	0.33 ± 0.025	0.67 ± 0.025	0.95 ± 0.025	0.05 ± 0.0025
60	0.37 ± 0.024	0.63 ± 0.025	0.95 ± 0.025	0.05 ± 0.0025
80	0.42 ± 0.023	0.58 ± 0.023	0.95 ± 0.023	0.05 ± 0.0025
100	0.46 ± 0.022	0.54 ± 0.022	0.94 ± 0.022	0.06 ± 0.03
120	0.49 ± 0.02	0.51 ± 0.021	0.92 ± 0.02	0.08 ± 0.04
136 (UCST)	0.74 ± 0.02		0.026 ± 0.013	

PSU50/DGEBA50-DGEBA50/AGE50				
T, °C	Left		Right	
	$\varphi_{PSU50/DGEBA50}$	$\varphi_{DGEBA50/AGE50}$	$\varphi_{PSU50/DGEBA50}$	$\varphi_{DGEBA50/AGE50}$
40	0.3 ± 0.028	0.7 ± 0.028	0.3 ± 0.028	0.7 ± 0.028
60	0.31 ± 0.028	0.69 ± 0.028	0.31 ± 0.028	0.69 ± 0.028
80	0.32 ± 0.028	0.68 ± 0.028	0.32 ± 0.028	0.68 ± 0.028
100	0.33 ± 0.028	0.67 ± 0.028	0.33 ± 0.028	0.67 ± 0.028
120	0.35 ± 0.028	0.65 ± 0.028	0.35 ± 0.028	0.65 ± 0.028
140	0.37 ± 0.028	0.63 ± 0.028	0.37 ± 0.028	0.63 ± 0.028
160	0.4 ± 0.028	0.6 ± 0.028	0.4 ± 0.028	0.6 ± 0.028
180	0.42 ± 0.028	0.58 ± 0.028	0.42 ± 0.028	0.58 ± 0.028

PSU45/DGEBA55-DGEBA55/AGE45				
T, °C	Left		Right	
	$\varphi_{PSU45/DGEBA55}$	$\varphi_{DGEBA45/AGE55}$	$\varphi_{PSU45/DGEBA55}$	$\varphi_{DGEBA45/AGE55}$
40	0.34 ± 0.03	0.66 ± 0.03	0.04 ± 0.02	0.96 ± 0.03
60	0.43 ± 0.03	0.57 ± 0.03	0.08 ± 0.03	0.92 ± 0.03
80	0.53 ± 0.03	0.47 ± 0.03	0.15 ± 0.03	0.85 ± 0.03
91 (UCST)	0.69 ± 0.03		0.31 ± 0.03	

Ternary phase diagram of system

T, °C	φ_{AGE}	φ_{DGEBA}	φ_{PSU}
40	0.085 ± 0.025	0.000	0.915 ± 0.025
40	0.1 ± 0.025	0.1 ± 0.025	0.8 ± 0.025
40	0.132 ± 0.025	0.198 ± 0.025	0.67 ± 0.025
40	0.38 ± 0.025	0.57 ± 0.025	0.05 ± 0.0025
40	0.17 ± 0.025	0.5 ± 0.025	0.33 ± 0.025
40	0.49 ± 0.025	0.5 ± 0.025	0.01 ± 0.005
40	0.205 ± 0.025	0.55 ± 0.025	0.245 ± 0.025
40	0.432 ± 0.025	0.55 ± 0.025	0.018 ± 0.025
40	0.01 ± 0.005	0.000	0.99 ± 0.025
120	0.17 ± 0.023	0.000	0.83 ± 0.023
120	0.1925 ± 0.023	0.1925 ± 0.023	0.615 ± 0.023
120	0.204 ± 0.023	0.306 ± 0.023	0.49 ± 0.023
120	0.364 ± 0.023	0.546 ± 0.023	0.09 ± 0.023
120	0.265 ± 0.023	0.5 ± 0.023	0.235 ± 0.023
120	0.485 ± 0.023	0.5 ± 0.023	0.015 ± 0.005
120	0.01 ± 0.005	0.000	0.99 ± 0.023
180	0.23 ± 0.022	0.000	0.77 ± 0.022
180	0.245 ± 0.022	0.245 ± 0.022	0.51 ± 0.022
180	0.35 ± 0.022	0.5 ± 0.022	0.15 ± 0.022
180	0.455 ± 0.022	0.5 ± 0.022	0.045 ± 0.022
180	0.48 ± 0.022	0.48 ± 0.022	0.04 ± 0.022
180	0.02 ± 0.01	0.000	0.98 ± 0.022
